# Prescription patterns and drug use among pregnant women with febrile Illnesses in Uganda: a survey in out-patient clinics

**DOI:** 10.1186/1471-2334-13-237

**Published:** 2013-05-23

**Authors:** Anthony K Mbonye, Josephine Birungi, Stephanie Yanow, Pascal Magnussen

**Affiliations:** 1 School of Public Health, Makerere University and Commissioner Health Services, Ministry of Health, Box 7272, Kampala, Uganda; 2 Senior Principal Research Officer, Division of Entomology, Uganda Virus Research Institute, Entebbe, Uganda; 3 Research and Development, Provincial Laboratory for Public Health, WMC 2B4.59, 8440 112th Street, Edmonton AB T6G 2J2, Alberta, Canada; 4 Centre for medical Parasitology, University of Copenhagen, Copenhagen, Denmark

**Keywords:** Malaria, Pregnant women, Appropriate treatment, SP, Uganda

## Abstract

**Background:**

Malaria is a public health problem in Uganda; affecting mainly women and children. Effective treatment has been hampered by over-diagnosis and over-treatment with anti-malarial drugs among patients presenting with fever. In order to understand the effect of drug pressure on sulfadoxine-pyrimethamine (SP) resistance in pregnancy, a sample of pregnant women presenting with fever in out–patient clinics was studied. The main objective was to assess prescription patterns and drug use in pregnancy especially SP; and draw implications on the efficacy of SP for intermittent preventive treatment of malaria in pregnancy (IPTp).

**Methods:**

A total of 998 pregnant women with a history of fever were interviewed and blood samples taken for diagnosis of malaria and HIV infections. Data were captured on the drugs prescribed for the current febrile episode and previous use of drugs especially SP, anti-retroviral drugs (ARVs) and cotrimoxazole.

**Results:**

Few pregnant women, 128 (12.8%) were parasitaemic for *P.falciparum*; and of these, 72 (56.3%) received first-line treatment with Artemether-lumefantrine (Coartem®) 14 (10.9%) SP and 33 (25.8%) quinine. Of the parasite negative patients (non-malarial fevers), 186 (21.4%) received Coartem, 423 (48.6%) SP and 19 (2.1%) cotrimoxazole. Overall, malaria was appropriately treated in 35.5% of cases. Almost all febrile pregnant women, 91.1%, were sleeping under a mosquito net. The majority of them, 911 (91.3%), accepted to have an HIV test done and 92 (9.2%) were HIV positive. Of the HIV positive women, 23 (25.0%) were on ARVs, 10 (10.9%) on cotrimoxazole and 30 (32.6%) on SP. A significant proportion of women, 40 (43.5%), were on both SP and cotrimoxazole. Age and occupation were associated with diagnosis and treatment of malaria and HIV infections.

**Conclusion:**

There is inappropriate treatment of malaria and non-malarial fevers among pregnant women in these facilities. This is due to non-adherence to the guidelines. Over-prescription and use of anti-malarial drugs, especially SP may have implications on resistance against SP for malaria prevention in pregnancy. The policy implications of these findings are to evaluate SP efficacy as IPTp; and the need to enforce adherence to the current clinical treatment guidelines.

## Background

Uganda has a high burden of malaria that contributes greatly to morbidity and mortality [1,2]. Malaria contributes to 40% of outpatient consultations and 20% inpatient admissions; and the most vulnerable are women and children [1]. The main component of the current malaria control strategy in Uganda is early diagnosis and effective case management. In Uganda and elsewhere, effective treatment has been hampered by the continuing over diagnosis of malaria and over-treatment with anti-malarial drugs among patients presenting with a fever particularly in rural areas where there is poor access to formal health facilities and self-treatment is the commonest form of care seeking [3,4].

Malaria in pregnancy is a major public health problem in malaria endemic areas [5-7]. The current policy in Uganda for treating malaria in pregnancy in the first trimester is to give quinine 10 m/kg. During the second and third trimester, ACT are recommended as follows: Coartem® (artemether 20 mg & lumenfantrine 120 mg) four tablets twice a day for three days [8]. For malaria prevention, the policy recommends two doses of SP as intermittent preventive treatment of malaria in pregnancy (IPTp). The rapid spread of *P. falciparum* parasites resistant to SP in malaria endemic areas poses a major threat to prevention of malaria in pregnancy[9-11]. There is now evidence of drug resistance to SP which may have implications for malaria control in pregnancy [12-14]. However SP is still the recommended drug for IPTp in Uganda [15,16]. Thus monitoring patients for resistant parasites during pregnancy is an important strategy to provide data for timely policy review. Recently WHO has issued guidelines on IPTp for countries in areas of moderate-to high transmission; IPTp with SP is recommended for all pregnant women at each scheduled antenatal care visit; and each SP dose should be given at least 1 month apart [17]. In order to effectively implement this guideline, it requires continuous monitoring of SP efficacy as IPTp.

In countries with a high HIV prevalence, IPTp with SP has been found to be less effective thus leading to a recommendation of increasing the number of doses of IPTp in pregnancy. Thus in areas of high transmission of *P. falciparum*, HIV-positive women are recommended to receive monthly doses of SP as IPTp rather than the 2-dose regimen [15,16]. The current policy in Uganda is to give trimethoprim-sulfamethoxazole (cotrimoxazole) prophylaxis for HIV infected patients. It has been shown that there is cross-resistance between trimethoprim and pyrimethamine at a molecular level [17]. It is possible that resistance alleles may emerge in response to selective pressure imposed by this treatment regimen [18-21]. In Uganda the policy doesn’t allow SP and cotrimoxazole to be given together; except when there is non-adherence to the guidelines.

Previous studies in Uganda have assessed prescription patterns of drugs in the public and private facilities. In one study, only 34% of the recommended doses of chloroquine followed the guidelines [22]. It has been further shown that only 30% of patients received treatment at a health facility within 24 hours of onset of symptoms [23]. In another study, polypharmacy was common in both private and public clinics and there was over-prescription of anti-malarial drugs and antibiotics [24]. Most recently, the commonly prescribed anti-malarial drugs in rural hospitals of Uganda were ACT and Quinine [25]. Despite this evidence, few studies have documented prescription patterns and drug use among pregnant women with fever visiting clinics in Uganda. This study aims to answer whether pregnant women with fever presenting at out-patient clinics get appropriate treatment, what drugs are prescribed and whether this is according to the guidelines.

Understanding treatment patterns for febrile illness among pregnant women in countries endemic for malaria is of public health importance for three reasons: 1) to gain an understanding of factors associated with treatment seeking among this vulnerable group and design interventions to increase access to effective treatment and prevention of malaria and other non-malarial fevers; 2) drugs given in pregnancy may have direct and indirect effects on the baby; 3) to understand the level of appropriate treatment since inappropriate drug use may lead to drug resistance. The main objective of this study was to assess prescription patterns and drug use among pregnant women with fever. Specifically the study assessed use of anti-malarial drugs during the current and previous febrile episodes; and use of SP and cotrimoxazole among HIV positive women. This article presents results on drug use among a sample of pregnant women presenting with fever at out-patient clinics in Uganda. Implications of the results and further areas of research are discussed.

## Methods

### Study area and population

The study was conducted in the malaria endemic district of Mukono, central Uganda. The total population of the district is 850,900 with an annual growth rate of 2.3% and consists predominantly of subsistence farmers of the Baganda ethnic group. The majority of the population, 88%, lives in the rural areas.

### Study design

A survey targeting pregnant women presenting with fever in out–patient clinics was carried out from January to November 2011. Inclusion criteria were: i) Actual fever or history of fever during the last 14 days, ii) temperature at least 37.5°C; this was taken using a digital thermometer iii) menstrual history and a clinical examination by a trained midwifes and clinical officers to confirm a pregnancy. A structured questionnaire was administered to all eligible consenting pregnant women with fever attending outpatient clinics over a period of 10 months. The questionnaire captured data on demographic characteristics, history of febrile illness, treatment received, HIV counselling and testing practices, history of taking SP, antimalarial drugs, ARVs and other drugs. Midwives working in two maternity units at Kawolo hospital and Mukono health centre IV conducted the interviews and patients who consented to give a blood sample were sent to a laboratory. The midwives were trained for 2 days on study procedures and they participated in the pre-testing and reviewed the tools before the study. Questionnaires were initially developed in English and translated into the local language (Luganda). The field coordinator and the research team supervised all aspects of data collection.

### Laboratory methods

Blood (4 mls) was taken by venepuncture from every patient who consented to the study and stored in EDTA tubes. Haemoglobin, HIV and malaria infections and were assessed. Smears were stained with Giemsa and malaria parasites were counted against 200 leukocytes and expressed as number of parasites per μl of blood assuming a standard leukocyte count of 8000/μl of blood. A blood smear was regarded negative after examining a minimum of 100 high power fields with no parasites seen. This is because the study area is known to be highly endemic for malaria thus the selection of this cut-off point. All buffers, solutions and stains were stored appropriately and cleanliness and sterility of equipment maintained. For quality assurance, all malaria positive samples examined in the field laboratory were re-examined at the reference laboratory at the Uganda Virus Research Institute. Discrepancy between the two readings was less than 5%.

HIV test was performed following the national algorithm on HIV testing. A blood sample undergoes three tests: the first is Determine® and if positive, it is confirmed by Statpak® if still positive by Unigold®. Results were recorded on forms and clients told the results.

### Statistical analyses

Data was entered and verified using Microsoft Access 2007 (Microsoft Inc., Redmond, Washington) and analysed using STATA version 11.0 (STATA Corporation, College Station, Texas). Univariate analyses were carried to get the proportions of drugs prescribed to women with malaria, non-malaria fevers and those who were HIV positive. Bivariate analysis was carried to assess factors associated with diagnosis and treatment of malaria and HIV infections. Proportions were compared using a chi-Square test and P-values <0.05 were considered significant. Appropriate treatment was calculated as the number of parasite positive pregnant women who received Coartem® plus parasite negative women who received no antimalarial drug divided by the total number of febrile cases. The sample size calculation was based on the proportion of pregnant women appropriately treated for malaria estimated at 25% [26]. The study aimed to detect a difference of 4% in this proportion at 80% power and 5% level of significance; and the number of patients to estimate this difference was 946. Allowing a 5% for non-response rate, the minimum number of patients was 993.

### Ethics

Ethical approval for the research was granted by review boards at the Uganda National Council of Science and Technology (Reference HS. 747). Written consent was obtained from all participating women.

## Results

### Patient characteristics

A total of 998 pregnant women presenting with fever were included in the study. The mean age was 23.9 years with a range of 14–42 years. The majority of women, 64.9%, were aged between 20–29 years, 33.7% had attained secondary education and 78.2% were married; while 47.7% were peasants earning from household agricultural activities (Table 1).

**Table 1:** Background characteristics of women pregnant women with fever attending outpatient clinics

**Patient characteristics**	**Frequency (%) (N = 998)**
**Age (years)**	
14-19	205 (20.5)
20-29	648 (64.9)
30-50	145 (14.5)
**Education level**	
None	101 (10.1)
Primary	491 (49.2)
Secondary	336 (33.7)
Tertiary (Technical/University)	70 (7.0 )
**Marital status**	
Single	168 (16.8)
Married	780 (78.2)
Cohabiting	2 (0.2)
Widowed	16 (1.6)
Separated	32 (3.2)
**Occupation status**	
Peasant /agriculture	472 (47.3)
Employed	132 (12.3)
Business/shop	276 (27.7)
Some activity that earns money	108 (10.8)

### Febrile illnesses among pregnant women and treatment received

Of the pregnant women presenting with fever, only 128 (12.8%) had parasitaemia. Of these, 72 (56.3%) received the first-line antimalarial drug, Coartem®. A large proportion 33 (25.8%) received quinine while 14 (10.9%) received SP (Fansidar®). Among pregnant women with no malaria parasites, 186 (21.4%) were given Coartem® and 423 (48.6%) Fansidar® (SP); 113 (13.0%) were given antibiotics. Appropriate treatment of malaria (patients with malaria receiving first-line drug (ACT) and patients without malaria receiving no antimalarial drug) occurred in 35.5% of pregnant women. The majority of patients 909 (91.1%) were sleeping under a mosquito net (Table 2).

**Table 2:** Prevalence of febrile illnesses among pregnant women attending outpatient clinics

**Patient characteristics**	**Frequency (%)**
**Duration of current febrile episode (days)**	**N = 998**
1**–**6 days	858 (86.0)
7–14 days	140 (14.0)
**Malaria parasite prevalence**	
Negative	870 (87.2)
Positive	128 (12.8)
**Treatment given to patients with confirmed malaria**	**N = 128**
Coartem	72 (56.3)
Fansidar (SP)	14 (10.9)
Quinine	33 (25.8 )
Paracetamol	1 (0.8)
Antibiotic	8 (6.2)
**Treatment of non-malarial fevers**	**N = 870**
Chloroquine	45 (5.2 )
Coartem	186 (21.4)
Fansidar (SP)	423 (48.6)
Quinine	32 (3.7 )
Paracetamol	52 (6.0)
Antibiotic	113 (13.0)
Cotrimoxazole	19 (2.1)
**Appropriate treatment of malaria**	
Parasite positive patients who received Coartem + parasite negative patients who received no antimalarial drug/total number of febrile cases	35.5%
**Malaria treatment and preventive practices**	**N = 998**
Proportion of patients who had taken an anti-malaria drug before this visit	313 (31.4)
Proportion of patients who slept under a net the previous night	909 (91.1)
Proportion of patients who sleep under a net every day	892 (89.4)

### HIV/AIDS testing and treatment received

The majority of pregnant women in this study were willing to have an HIV test done, 911 (91.3%) (Table 3); while 361 (36.2%) had had a test prior to this visit. Adolescents were less likely to have had a previous HIV test, 88 (13.9%) compared to 547 adults (86.1%), *P* = 0.0001 (data not shown). Among the pregnant women who had an HIV test, 92 (9.2%) were HIV positive. A quarter, (25.0%) of HIV positive women were on ARVs, while 30 (32.6%) of them were taking cotrimoxazole. Forty HIV positive women (43.5%) were taking both cotrimoxazole and SP (Table 3).

**Table 3:** HIV/AIDS prevention and treatment practices

**Patient characteristics**	**Frequency (%)**
**Patients willing to have an HIV test**	**N = 998**
Yes	911 (91.3)
No	87 (8.7)
**HIV testing results**	
Positive	92 (9.2)
Negative	906 (90.8)
**Treatment/prevention of HIV/AIDS**	**N = 92**
HIV positive patents on ARVs	23 (25.0)
HIV positive patents on cotrimoxazole	10 (10.9)
HIV positive patents on SP	30 (32.6)
HIV positive patents on cotrimoxazole and SP	40 (43.5)

### Factors associated with diagnosis and treatment of malaria and HIV

We explored factors associated with diagnosis and treatment of malaria and HIV. Younger women were likely to test positive for malaria, mean age 22.3 years versus 24 years for negative women, *P* = 0.0002; and receiving anti-malarial treatment, *P* = 0.02. Similarly occupation, *P* = 0.004; and tribe, *P* = 0.02 were associated with receiving anti-malarial treatment. HIV positivity was associated with age (mean age 23.7 years for HIV negative versus 24.9 years for positive women, *P* = 0.02); and access to ARVs (mean age 23.9 years for HIV negative versus 25.9 years for HIV positive women, *P* = 0.02). Education, marital status and religion were not associated with diagnosis and treatment of malaria and HIV infections in this population.

## Discussion

This study shows that there is inappropriate treatment of malaria among pregnant women with fever attending outpatient clinics. Although ACT are the first-line anti-malarial drug, many patients with confirmed malaria were not given ACT but SP. This may lead to parasite resistance to SP. Similarly, inappropriate use of ACT in pregnant women with non-malarial fevers may also lead to resistance against these drugs. These results will contribute to the design of a further study to evaluate the efficacy of SP as IPTp in Uganda.

A recent study in health facilities in Uganda found out that there were constraints to parasite diagnosis like lack of microscopes and RDTs, inadequate personnel and infrastructure. This scenario impacts on appropriate treatment of malaria and other diseases. Similarly a study in Jinja, a neighbouring district in Eastern Uganda showed that 85% of self-reported febrile illnesses were treated with antimalarial drugs [27].

The present results compare with those from a study carried out in South-Western Uganda in an area of low malaria endemicity which found high prescription rates for antibiotics in febrile patients, the majority of whom were children aged less than 5 years, and concluded that testing negative for malaria increases the use of antibiotics [25]. The present study, however, shows that antibiotic prescription among febrile patients was low and this is likely because these were adult patients and the risk of pneumonia could have been low. It is also possible that health workers may not be thinking of common diseases in pregnancy that may require antibiotics. A diagnosis and treatment alogorithm for helping health workers to treat malaria and other common diseases is recommended and could include confirming pregnancy and the gestation period; checking urine for infections, HIV testing, screening for lung infections and history of anti-malarial drugs especially of SP.

Our study shows that access to ACT is high, consistent with findings from another Ugandan study that the most commonly prescribed antimalarial drugs are artemether-lumefantrine and quinine with a relatively high adherence to the new antimalarial treatment policy [28]. This contrasts with an earlier study conducted across six African countries that showed poor access to ACT in Benin (10%), DRC (5%), Madagascar (3%), Nigeria (5%), Uganda (21%) and Zambia (21%) [26]. Increased access to ACT in Uganda is attributed to a recent government initiative through the Global Fund that supports local manufacturing of ACT and ARVs. Access and use of ARVs is however poor as noted in our results due to inadequate laboratory services to analyze CD4 count, a pre-requisite to start patients n ARVs.

In interpreting the results it is worth noting that this study targeted pregnant women with fevers reporting in outpatient clinics thus the drug use and prescription patterns in other groups were not included. Clinical examination was relied to estimate the gestation period and thus could have missed pregnancies in the first trimester. In addition, the study was limited in the capacity to identify the causes of non-malaria fevers. A study carried on Thai-Burmese border found that non-malaria fevers in pregnancy were mainly scrub and typhus fevers [29]. Since such infections are not common in Uganda, it would be of interest to have a study to document the causes of non-malaria fevers among pregnant women in Uganda. This study looked at fever in pregnancy; however diagnosis of malaria in pregnancy is problematic since most pregnant women in highly endemic areas have immunity, are asymptomatic and peripheral blood may not show parasitaemia [30]. Thus this study may have under-estimated the magnitude of malaria in pregnancy.

The implications of these results are to ensure that health workers adhere to laboratory test results and prescribe according to the treatment guidelines. This will ensure appropriate treatment and avoid indiscriminate drug use potentially leading to drug resistance. Printing and distributing treatment guidelines supported by technical supervision are urgently needed to address this situation. Further areas for research include evaluating the efficacy of SP as IPTp and the review of the guidelines for IPTp among HIV positive women since interaction of SP with cotrimoxazole could hamper the effectiveness of SP. There is also need to strengthen pharmacovigilance of antimalarial drugs to enable timely review of policies.

## Conclusions

There is inappropriate treatment of malaria and non-malarial fevers among pregnant women in these facilities. This is due to non-adherence to the guidelines. Over-prescription and use of anti-malarial drugs, especially SP may have implications on resistance against SP for malaria prevention in pregnancy. The policy implications of these findings are to evaluate SP efficacy as IPTp; and the need to enforce adherence to the current clinical treatment guidelines.

## Competing interests

The authors declare that they have no competing interests.

## Authors’ contributions

AKM conceived the study; AKM, JB, SY and PM participated in the design, implementation, coordination of the study and drafting the manuscript. All authors read and approved the manuscript.

## Pre-publication history

The pre-publication history for this paper can be accessed here:

http://www.biomedcentral.com/1471-2334/13/237/prepub
